# Clinical Review and Hospital Reports

**Published:** 1840

**Authors:** 


### Clinical Review.

### GUY'S HOSPITAL.

GUY'S HOSPITAL REPORTS. No. X. APRIL, 1840. Edited by GEORGE H. BARLOW, M.A. & L.M. Trinity College, Cambridge; Inceptor Candidate of the Royal College of Physicians, and Physician to the Surrey Dispensary: and JAMES P. BABINGTON, M.A. Trinity College Cambridge; Member of the Royal College of Surgeons. London: S. Highley.

The present Number is not as large in size as sundry of its predecessors, but perhaps it is not less than them in value. A guess may be made on the latter, from a glance at the contents of the volume.

It consists of:—1, Some Observations tending to demonstrate the Dependence of Vascular Organization upon Physical Causes, by Sir Anthony Carlisle, F.R.S. (with Plate)—2, Observations on the Existence of certain Elements of the Milk in the Urine during Utero-Gestation; and on the application of this fact to the Diagnosis of Pregnancy; by Golding Bird, M.D. F.L.S.—3, On the Common Action of the Auriculo-Ventricular Valves; by T. Wilkinson King, (with Plate). 4. On the Surfaces of Contact, or Attrition, on the Valves of the Heart; by T. Wilkinson King, (with Plates)—5. Account of the Fibrous Structure of the Sub-serous Membrane of the Aorta; by Norman Chevers, M.D. (with Plates) —6. On the Morbid Consequences of Undue Lactation; by Samuel Ashwell, M.D.—7. Practical Hints on the Treatment of Stricture; by Bransby B. Cooper, F.R.S.—8. Cases of Excision of the Elbow-Joint; by C. Aston Key.—9. The History of a Gun-Shot Wound, in which the Patella was carried away, and the Knee-joint completely laid open: successfully treated by W. Ward of Huntingdon. Communicated by Mr. Bransby Cooper.—10. Case of the Dislocation of the Shoulder-joint, with Fracture of the Humerus. In sequel to Sir Astley Cooper's Paper on the same subject. By J. A. Hingeston. Communicated by Mr. Key.—11. On the Rapidity of the Circulation in the Lymphatic System; by T. Wilkinson King.—12. Cases and Observations illustrative of Renal Disease accompanied with the Secretion of Albuminous Urine. Memoir the Second. By R. Bright, M.D. F.R.S.—13. On the Proportion of Urea in certain Diseased Kidneys; by G. O. Rees, M.D. F.G.S.—14. Cases of Albuminous Urine, illustrative of the Efficacy of Tartar Emetic, in combination with other Antiphlogistic Remedies, in the acute form of that Disease; by George H. Barlow, M.A. & L.M.

We shall take the practical papers first, as, perhaps, from their intrinsic value, they deserve. We shall commence with that on the:—

### MORBID CONSEQUENCES OF UNDUE LACTATION. By S. ASHWELL, M.D.

Dr. Ashwell thinks that the subject has hardly received a fair share of attention. He starts by laying it down as a canon, that *exhaustion*—generally attended by symptoms of re-action, but occasionally by depression, so extreme as almost to conceal any such effort—constitutes the prominent, the essential feature when lactation has become a disease. Anæmia, with irritability and universal pallor, are as apparent as in chlorosis: of course, in different degrees. In some instances there is distressing debility: in other, and less serious cases, there is only trifling anæmia, and proportionately slight pallor. Local congestion, also, as it is the result of an irregular distribution of the blood, may partially modify the anæmia and pallor, by producing, in certain organs, a temporary but morbid energy, and, by fulness of the capillaries, a less pallid and unhealthy aspect of the surface. Still, exhaustion is the permanent morbid state, associated with undue suckling.

He is of opinion that it may be proved :—

First, That lactation to be morbid need not be long: evil consequences may ensue soon after its commencement; occasionally, within a few weeks; more frequently within a period protracted beyond nine months.

Secondly, That organic lesions may, although very rarely, result from undue suckling.

And, Thirdly, That weaning the child is generally indispensable to the cure—the remedy, without which all others will be inefficient.

The majority of women, no doubt nurse well for a reasonable time, but if, from over-nursing, or from nursing with a constitution naturally weak, or artificially enfeebled, it begins to suffer, a train of symptoms is induced which Dr. Ashwell describes.

"Amongst the earlier symptoms of failure are, a heavy, dragging sensation in the back and loins, and directly between the scapulæ, when the child is at the breast; and a feeling of peculiar sinking and emptiness at the pit of the stomach, and over the whole abdomen, for hours afterwards. Inquire particularly, and you will discover, what is often anxiously concealed, that the milk is scanty in quantity, and with difficulty secreted ; and that without long intervals, scarcely any fresh supply would be furnished. At this point, much might be done. If weaning entirely were practised, the symptoms would soon disappear: or if only partially adopted (by the child being judiciously fed, and the mother's rest at night secured, instead of being continually broken), lactation might be safely continued ; as the appetite, digestive powers, and strength of the parent would be thereby improved. But the attempt to nurse is often persevered in, without these advantages ; and the morbid results are soon aggravated. Together with an excitement or depression of mind, there is a proneness to hysteria ; the pulse is quicker than natural, and easily compressed ; the muscular system is weakened; the appetite is nearly destroyed, or it is at least fastidious and unhealthy; the bowels are either constipated and flatulent, or painfully griped, and slightly purged; there is headache, or giddiness, with impaired vision ; pain between the shoulders, or in the sides, below the cartilages of the false ribs : now, but especially if the suckling be continued, there is swelling of the ancles, œdema of the face, and frequent palpitation."

Should these symptoms grow more intense, we have then a severe and well marked case. Particular symptoms not unfrequently attract the principal attention. Impaired appetite is one of these—palpitation another—dimness of vision a third ; yet all are functional, or, at least, are not necessarily otherwise.

Dr. Ashwell next alludes to *occasional complications* of morbid lactation.

1. *Profuse Menstruation, Menorrhagia, and Leucorrhœa.*—There is nothing surprising in the occurrence of these. Dr. Ashwell rather thinks that they prepare the way for organic diseases of the womb.

2. *Functional Amaurosis.*—This, accompanied with congestion of the conjunctiva, is common. The patient is alarmed, yet,—

"In the greater number of cases, prompt weaning will alone remove the affection : still, it may be necessary repeatedly to apply small blisters near the eye, and absolutely to forbid its employment. Improved diet, country and sea air, exercise out of doors, iron and quinine, are important remedial auxiliaries. Nor is it unimportant that quickly-recurring pregnancy should, if possible, be avoided. I have known several instances where, during a pregnancy immediately succeeding the exhaustion of over-nursing, the eye has been almost constantly in a state of 'blood-shot' or congestion, and the sight excessively imperfect. Months, and even years, sometimes elapse, where able treatment has done its best before distinct and strong vision is re-acquired. Specks, and slight ulcerations of the cornea, are occasionally connected with the exhaustion and irritability of nursing. In all these cases, provided there be no serious organic change, the sufferer may be encouraged certainly to expect the restoration of this most invaluable faculty."

3. *Jactitation.*—Dr. A. has seen several instances of this. In one poor woman, an out-patient of Guy's Hospital, the seizures always occurred after she had nursed for three or four months; and they were so violent, that she was compelled to lay down her baby when they occurred, lest she should let it fall. In another young and hysterical patient, who had borne children very quickly, there was, during lactation, an almost-continual and slight twitching pretty universally throughout the extremities, but especially of the face. In both, weaning was always necessary before the sixth month, more on account of leucorrhœa and general irritability, than for the jactitation.

4. *Epilepsy* has been noticed as a consequence of lactation, and Dr. Ashwell has seen fits difficult to be distinguished from the epileptic, result from it.

5. *Insanity.*—This commences by peculiarity of sentiment or temper, and "is plainly evinced by pertinaceous adherence to an opinion once formed, however erroneous; and scarcely at all more strikingly displayed than in a determined opposition to any advice having for its end an entire or even a partial weaning. In this early stage, the further advance or the protracted continuance of the malady might probably be thus prevented: but, instead of weaning, larger quantities of porter or wine, with animal food, are most improperly resorted to. Still the desired supply is not obtained. The stomach has been already weakened; and as it is scarcely able to bear a diminished diet, fever and indigestion are only apparent and temporary, not real strength, must be the consequence of this increased supply. Together with a continued sparing secretion of milk, the symptoms already described are aggravated. The insanity becomes positive and acute, the pulse quick and sharp, the skin parched, and the whole system deranged. The conduct of the patient is no longer doubtful: her actions are often violent; and, without personal restraint, serious, perhaps fatal injury might be inflicted on herself, and those around her. I agree, however, with Dr. Locock, that the aberration of undue suckling is rarely of this serious kind, excepting where generous diet and wine are injudiciously administered: more commonly it shews itself in weakness and absurd ideas, in whim and caprice. If weaning and careful treatment be even now adopted, the symptoms often subside easily and quickly: while in other cases, where probably a disposition to insanity exists hereditarily, the disease is of longer duration, requiring seclusion and confinement for its cure."

Dr. Ashwell has never known an instance of permanent insanity from lactation. He is satisfied of a resemblance between this and puerperal mania. Both are disposed to recur in after-confinements. In the greater number of examples of puerperal insanity, a modified antiphlogistic treatment only, comprising small local bleedings, cordial aperients, and particularly sedatives, with mild nourishment and tonics, is most successful; and the same may be said of the insanity from over-lactation. But puerperal mania, (and the explanation is obvious) is very much the more frequent.

6. *Undue Suckling may, although rarely, induce organic change in the Brain, Lungs, and Uterus.*

Dr. Ashwell believes this to be a fact. He first alludes to *Headache.* "So long," he says, "as it is general, not very severe and transient—so long as it does not recur periodically, with marked premonitory symptoms—it may be viewed as comparatively free from risk. But if it be dreaded on account of the permanent uneasiness which it has already produced, or from its intensity and acuteness; if it seize on one part of the head, and remain fixed there; if its paroxysm be preceded by rigors, and if the pain never entirely subsides; more especially if there be partial paralysis, mental peculiarity, or forgetfulness approaching to imbecility; or any other anomalous symptom indicative of deranged nervous action, for instance, an unusual affection of the eye, such as, double or impaired vision; or of the auditory nerve, injuring the hearing, or rendering it excessively and painfully acute; or if there be impeded deglutition; then danger exists, and a softened, or otherwise structurally altered condition of the brain, may be feared. Still, if weaning has not been adopted, it ought yet to be urgently enjoined."

*Phthisis* may be induced, more particularly if a previous tendency to tubercles existed.

The *uterus* may, he thinks, undergo *organic changes* too.

*Treatment.*—Where the symptoms of exhaustion are slight, a better diet, a careful regulation of the bowels, a tonic treatment, and, above all, diminished suckling, will often avail. The child should be fed two or three times within the twenty-four hours, and unbroken sleep should be secured to the mother. But a continuance of the debility, or the aggravated prevalence of one or more of the symptoms already enumerated, will plainly indicate the necessity of entire weaning. If the child be purged or become gradually emaciated, it will corroborate the importance of the step.

If organic disease threatens, the treatment must be such as would be appropriate to it. The convalescence of such patients is generally protracted and difficult, years sometimes elapsing prior to recovery. Nor can it be too forcibly recommended, that suckling should be abandoned, if a fresh pregnancy succeed very quickly. The symptoms are often rendered worse by gestation, and invariably by a renewed lactation. Iron, chalybeate waters, country and sea air, travelling and exercise, are most important auxiliaries.

### PRACTICAL HINTS ON THE TREATMENT OF STRICTURE. By BRANSBY B. COOPER, F.R.S.

Mr. Cooper, properly enough, disputes the muscularity of the urethra, anterior to the bulb, and the existence of "spasmodic stricture" in that part of the canal. We must say that we never saw anything which could be compared to spasm before the instrument arrived *at* the bulb. Mr. Cooper indeed remarks, that the error has doubtless arisen from the circumstance, that the phænomena attending sudden obstruction of the urethra are very similar, but not identical, with the contraction of muscular fibre. When a portion of the erectile tissue of the corpus spongiosum is suddenly distended from some morbific cause, in the same manner as the whole of it is naturally distended under venereal excitement, the urethra is partially contracted, as, in the latter case, it is in its entire course; and this contraction, from its suddenness, is attributed to the action of muscular fibre. The fact is, that any cause which produces a partial determination of blood to the corpus spongiosum necessarily produces also a sudden and partial obstruction in the urethra. The fallacy of regarding these obstructions as spasmodic contractions has doubtless been strengthened by the observation, that they are relieved by the same remedies as the latter—by bleeding, purging; by nauseating medicines, the warm bath, &c. But we must repeat that we never saw anything comparable to spasm, save in those parts of the canal which muscle surrounds, and where, indubitably, spasm occurs.

Mr. Cooper dwells, and not more earnestly than the occasion warrants, on the necessity of combining constitutional means with the use of instruments in the management of stricture. And he is anything but an advocate for force. The object, in his opinion, ought not to be, to force the instrument through the obstruction, but to press it upon it, or into its substance, where the nature of the latter admits of it, so as to alter the action going on in it, to induce inflammation, softening-down, and removal by absorption; in short, to employ, the instrument "*arte non vi,*" as was recommended by the great Dupuytren.

"Where the stricture is irritable, as is indicated by its tendency to bleed, and by the peculiar diathesis of the patient, recourse should be had to opiates, the warm bath, and caustic bougies. In cases where a disposition to spasm is observed, bleeding, opium, and belladonna injections will be found useful. Where the stricture, from its thickness, resists the gentle application of bougies, I have been very successful in rendering it permeable by injecting warm water into the urethra from a long canula, to which a syringe is attached; and by careful, continued and gentle pressure with this instrument, I have almost invariably succeeded in effecting a radical cure. In cases of irritable stricture, which I have described above, sedatives should be administered to allay the constitutional irritation; leeches may be applied to the perineum, as also belladonna fomentations, and the *gentle* use of the bougie may be recommended; but should the application of this be followed by bleeding and great pain, a very small piece of potassa fusa may be passed down to the stricture, and will be found to be an almost infallible remedy for the symptoms of irritability."

Mr. Cooper appears inclined to patronize the caustic bougie. We cannot help thinking, however, that the caustic bougie is very rarely necessary. Gentleness, perseverance, and judicious management will triumph over the great majority of strictures.

"The forcible introduction of a catheter or sound into the bladder is only justifiable in a few cases; and never where it cannot be effected without great violence, and without risk of laceration. Where the patient presents severe symptoms of retention, requiring immediate relief, such as great distention of the bladder, great constitutional irritation, and violent pain, an attempt should be made to pass a catheter; and if this instrument can be brought to a right angle to the position of the recumbent patient, and then, and not till then, becomes checked in its progress to the bladder, it is plain that the obstruction is situated at the membranous part of the urethra, where the operator may safely use force, if he apply it judiciously, and by depressing the handle of the instrument: for the risk which would be incurred in other portions of the urethra by such a proceeding is here in a great measure precluded by this portion of the canal being firmly connected to the surrounding parts of the deep fascia of the perinæum, and by the instrument itself being here guided and protected in its course by the ossa pubis.

But even in these cases, it is impossible to describe the *degree* of force which it is proper to resort to: language is inadequate to express the infinitely various exigencies of particular cases: the extent to which force should be carried can only be prescribed by the experience and tact of a practised surgeon, who has a perfect knowledge of the anatomy of the parts. Some surgeons would in such cases recommend force sufficient to thrust the instrument into the bladder: but I am confident that this is bad practice, and that it is much safer to cut down upon the membranous portion of the urethra than to risk the laceration of the canal, the perforation of the prostate gland, or the forcing of the instrument into the rectum; for all these are casualties to which violent treaters of stricture are liable; and I have known them to occur frequently. A stricture lacerated by this operation is, moreover, almost certain to recur; nay, to become more impervious than ever, as soon as the instrument is no longer regularly introduced: so that not only are the above risks incurred, but no ultimate good is effected.

In illustration of my views respecting those peculiar circumstances which justify the use of force, I will relate a case in which I preferred adopting this method of relief to an operation. A man was admitted into Guy's Hospital with a stricture, which, from its origin, might be called traumatic; but which in reality was not so, as the accident to which it was to be referred had not caused laceration of the urethra, but only inflammation of the surrounding parts, consequent thickening, and diminution of the calibre of the canal, as from ordinary causes.—I may here observe, that accidental contusion may produce stricture in any part of the canal; while from disease, as has been stated above, it most frequently occurs either in the bulb or membranous portion. The peculiar symptom in this man was the constant dribbling of his water from him; so that he did not appear to be suffering from retention of urine, though, in fact, there were present many urgent symptoms of that state, viz. pain in the region of the bladder, pain in the loins, numbness of the thighs, highly ammoniacal urine containing mucus in considerable quantity. There is no symptom occurring in cases of permanent stricture which more imperatively requires attention than this involuntary dribbling of urine; which, though it precludes the necessity of an operation for the immediate relief of a patient, as in cases of perfect retention, still permits the most dangerous symptoms arising from dysuria to go on slowly, but surely, to the destruction of the patient. Convinced of this, and finding, on a minute examination of my present patient, from the smell of the urine, the distention of the bladder, and the tenderness in the hypogastric region, that the case was more urgent than was apparent on a cursory inspection, I at once attempted to pass a full-sized catheter (No. 8) into the bladder. It penetrated without any difficulty, as far as the posterior part of the membranous portion of the urethra, but there encountered a sudden and definite obstacle to its further progress: to break down this, I used force, to which it at length yielded, and the instrument went into the bladder with a jerk. Now the circumstances which in this case induced me to resort to force, were as follows: In the first place, the patient had so long laboured under aggravated symptoms of permanent stricture, that his state was already very precarious. Secondly, though the actual retention of urine was not, as usually happens, the most urgent symptom, a free passage into the bladder was nevertheless indispensable to his cure. Then, again, the prostate gland was healthy, as was proved by examination *per rectum*, and the position of the obstruction was favourable to the application of force; for, guided by the forefinger of my left hand in the bowel, and making the deep fascia of the perinæum a fulcrum for the instrument, I was enabled to direct the latter with precision into the bladder, and at any rate, with as little danger as would attend the cutting into the membranous portion of the urethra, or the destruction of the stricture by caustic; one or other of which modes of relief I must otherwise have had recourse to. I hold, then, that where the symptoms are urgent, and the stricture is situated posteriorly to the deep fascia of the perinæum, force may be employed with propriety; but that where the stricture is at the bulb, though the symptoms are not more severe, an operation should be performed. In such cases as I have just described, however, force, as has been before observed, should only be used to the degree which the experience of the surgeon forbids him to exceed: where it remains without effect, and delay is admissible, warm baths, enemata, bleeding, or opium with small doses of tartarized antimony, may be tried as constitutional remedies, together with such local means as injection of solution of belladonna, or friction with mercurial or iodine ointment. Where the symptoms are urgent, and the surgeon, even after these pharmaceutical remedies, again attempts in vain to pass the catheter—always recollecting that much less force should be employed where the stricture is situated anteriorly to the membranous portion than in strictures of that region itself—he should immediately propose the operation of opening the membranous portion of the urethra."

Mr. Cooper advises this to be performed in the following manner. The patient being placed in the same position as for lithotomy, an incision, about two inches long, is to be made in the raphe of the perineum, dividing the superficial fascia. After the incision has been made, the second step of the operation is to pass the forefinger of the left hand into the upper part of the wound, directing it towards the arch of the pubes; when the urethra will be readily felt, especially if the patient be desired to strain, as in the attempt to make water. An incision is then to be made into this distended and fluctuating canal; through which opening, a female catheter is to be passed into the bladder, and the urine drawn off.

"The question now arises, in what manner a complete cure is to be effected. This depends upon the situation of the stricture. If, as is usually the case, it is behind the scrotum, the following means should be employed. The urine having been drawn off, as described, through a female catheter, a male catheter should be passed through the penis down to the stricture: its point should then be felt for, with the finger, in the incision which has been made in the perinæum; and will be perceptible through the thickness of the stricture, the distance between it and the finger being, of course, the depth of the adventitious growth which constitutes the stricture. This must next be divided by the knife; and the male catheter may then be passed on into the bladder, through the opening which had been made for the introduction of the female catheter. The instrument is afterwards to be kept in the bladder, and the patient put to bed. I decidedly recommend that the catheter should be left in the bladder, though this practice has been condemned; for without it, the divided stricture would certainly close again, and become more permanently firm than ever, the urine would be extravasated into the perinæum, and the patient would be subjected to these additional sources of irritation, if nature did not convert the perinæal opening into a permanent fistulous passage, which is sometimes effected by the formation of a new mucous lining, admitting the passage of the urine with impunity. Where the stricture is situated in the penis anteriorly to the scrotum, it is not safe to divide it with a knife, from the difficulty of afterwards closing the wound: and therefore it is better, in that case, only to draw off the urine through an incision in the membranous part of the urethra, by means of the female catheter, and to treat the stricture afterwards by passing bougies, in the same way as where immediate relief is not required."

### Cases of Excision of the Elbow Joint. By C. Aston Key.

Mr. Key has performed excision of the elbow-joint in three cases. The first case was detailed in the first volume of the Reports. It succeeded in a great degree. The two present cases have been fortunate, and are calculated to encourage resort, in proper circumstances, to the operation. Mr. Key observes:—

"The most favourable cases for the operation, so far as my limited experience has gone, appear to be those in which the disease has had its beginning in the articular surfaces. The substance of the bones is usually sound, and a healthy granulating process quickly follows the excision of the diseased surfaces. If the ulceration has spread from the cancelli to the articular cartilage, a sound surface cannot be ensured by the saw; and the healing of the parts is slow, or retarded by repeated exfoliation of small pieces of bone.

Hence the operation will probably succeed better in the adult than in the young subject. In the latter, articular disease often, probably in most cases, arises in the texture of the bone beneath the articular cartilage; and spreads by ulceration towards the cavity of the joint, which it opens and renders the seat of an acute suppurative action. The extent of disease in the bone is not easily defined, and the cut surfaces will probably not be in a perfectly healthy condition. The state of constitution, too, that gives rise to disease of bone, is unfavourable for an operation, much more so than the lowest cachexia induced by simple joint-disease. In regard to this operation upon children, however, I have no experience to offer, as I have hitherto performed excision upon adults only : but even viewing the operation as less promising in the young, I should not hesitate to perform it on a child, in lieu of amputation.

The disease for which the operation is required, begins not unfrequently in the lesser sigmoid articulation of the ulna on the head of the radius, from a twist or strain of the fore-arm acting on the coronary ligament. In the early stage, rotation is the motion most limited ; and the other signs of inflammation are confined to the radial part of the joint. The larger cavity of the ulna next becomes the seat of morbid action ; and gradually the whole surface of the joint is involved."

The cases themselves do not seem to require insertion.

THE HISTORY OF A GUN-SHOT WOUND, IN WHICH THE PATELLA WAS CARRIED AWAY, AND THE KNEE-JOINT COMPLETELY LAID OPEN : SUCCESSFULLY TREATED BY MR. WARD OF HUNTINGDON.

On the evening of Nov. 2, 1838, Mr. Ward was called to Mr. E. M. who had received a gun-shot wound from a fowling-piece in the right knee. Mr. Ward met Mr. Abbott, surgeon, of Cambridge.

" The contents of the gun had struck the patella on the outside of the knee, carrying away the whole of that bone, except a small, solid, triangular, portion which still remained attached to the ligament: there was a nearly circular wound of the integuments, completely exposing the joint, and sufficiently large to admit my whole hand into the joint between the tibia and femur ; but the cartilages of those bones appeared uninjured."

The consultants determined to attempt to save the limb. The patient was placed on his back, with the knee slightly flexed : a large poultice applied to the wound, and a full dose of opium given. No unfavourable symptoms, either local or constitutional, occurred during the progress of the case, nor was his pulse even in any degree accelerated. An anodyne at bed-time for a few nights, and occasional aperients, were the only medicines required. Poultices were con-continued until granulations began to arise; after which (the remaining small portion of patella having been removed) the surface was dressed with lint dipped in oil, and strips of adhesive plaster were applied in various directions, to assist in approximating the edges of the wound. On the 21st of Jan. 1839, the wound being quite healed, Mr. M. was able to dress himself, and sit up in a chair. In a short time, with the aid of a suitable splint and bandage, he went upon crutches ; and in the middle of March, he rode to Mr. Ward's house, six miles, on horseback. The cicatrix is now firm, and there is considerable motion of the joint ; so that Mr. M. can not only walk well without a stick, and even run without much inconvenience, but in November last Mr. Ward saw him dancing quadrilles at a ball.

Mr. Bransby Cooper appends some observations to this case. Among others, he makes the following :—

" As a joint, whose physical and functional characters have been altered by disease, may be exposed and partially or wholly removed with impunity, so would recent experience appear to encourage the expectation that, after an articulation has been laid open by violence, nature, aided by judicious treatment, may speedily produce such an alteration in its conditions, as shall prevent, or at any rate diminish, that excessive and almost fatal degree of constitutional irritation which has generally been considered the inevitable consequence of such lesions. In the cases of extensive wounds into joints, nature does not seem to attempt to close the synovial capsule by adhesive inflammation and thus re-convert it into a closed secreting membrane; but a granulating surface shuts up the wound into the articulations, and the joints are soon placed in a very similar condition to one which, after having been the subject of a violent inflammatory action from disease, is progressing towards a favourable termination.

In punctured wounds, the same action does not seem to follow; but the synovia continues to be secreted, oozes from the wound, intercepts nature's efforts at reparation, and induces a high degree of inflammatory action, which frequently leads to suppuration and death. In the latter cases, also, there is not the same degree of prostration as where an extensive wound lays upon a large joint, which accident is generally followed by faintness, almost approaching to collapse,—a condition most favourable, and likely to prevent subsequent high inflammatory action, by allowing the membrane, when in a passive state, to be submitted to the influence of external and apparently noxious agents.

The high degree of constitutional irritation which follows the infliction of a small punctured wound into a joint may also, in some measure, be attributable to the absence of that alarm which is created by a large wound; so that the same precautionary measures, rest and remedies, are not had recourse to. This supposition seems verified by the fact, of the operation for the removal of loose cartilages from the synovial membranes being safely performed by skilful surgeons;—skilful, I mean, not only in the manipulation employed for the removal of the extraneous substance, but also in the judicious preparation of his patient."

Much of the danger, we fancy, of punctured wounds of joints results from the liability to suppuration, and to the matter when formed being *shut up*. In large wounds of the articulations this can hardly occur. We feel disposed to doubt the influence of the primary shock in preventing inflammation. Mr. Cooper's remarks are deserving of attention.

### Case of Dislocation of the Shoulder-joint, with Fracture of the Humerus. In Sequel to Sir Astley Cooper's Paper on the same subject. By J. A. Hingeston.

Mr. P——, aged 63, of a spare habit, and in declining health; the muscular structure being slender and feeble, slipped down some cellar stairs. He fell with the left arm stretched out, and at the same time received a blow on the back of the humerus; by which violence, it would seem, the arm was knocked forward; while the head of the bone was pulled backwards by the scapular muscles, the scapula itself being the fulcrum. The head of the humerus was in this manner at once both fractured and dislocated, the fracture traversing the anatomical neck of the humerus.

*Signs.*—A falling down of the left shoulder; empty glenoid cavity; arm close to the side; the patient supporting the elbow of the injured arm in the opposite hand; the palm of the hand of the injured limb lying flat against the stomach (half-supine). On looking at the patient a short distance off, there was a visible protuberance under the clavicle, elevating the pectoral muscles; the axis of the limb, however, not being that of a dislocated shoulder. On examining the shoulder by touch, the head of the humerus was easily perceptible to the fingers of the operator, both under the clavicle and in the axilla. By placing the knee under the axilla, and making the usual extension for reducing dislocation, the operator, while in the act of pressing down the elbow, felt the grating of a fracture under the hand that grasped the shoulder-joint. Then, by grasping the shoulder and dislocated head of the humerus with the fingers of the one hand, and at the same time (the knee being still in the axilla) grasping the elbow with the other hand and jerking the shaft of the humerus upwards and outwards, the grating of the fracture became perceptible, frequent, and unequivocal. On the operator removing his hands and not interfering the least with the injured limb, but steadily looking at it in front, he could observe (as the patient was very thin) a manifest incongruity between the site of the dislocated head and the axis of the pendulous shaft of the bone. On searching at the top of the bone close to the dislocation and fracture, the fingers of the operator could be slipped into the fissure caused by the fracture between the separated ends of the bone.

The *treatment* was simply that of supporting the limb in a sling; and of the application of poultices, fomentations, &c. to assuage pain.

As the case proceeded, there was to be remarked a difficulty of supination and extension of the fore-arm, an inability to raise the elbow from the side, and a partial filling up of the glenoid cavity. At this period (Dec. 16th) there were all the signs of simple dislocation, with fixture of the fore-arm at a right angle across the body. The case might readily be taken for one of unreduced dislocation. On the 21st of December, the condition of the limb was as follows:—

First, The head of the humerus was broken off, and lying under the outer end of the clavicle in front of the coracoid process of the scapula.

Secondly, The glenoid cavity was empty, but somewhat filled up anteriorly by the head of the humerus resting on the anterior edge of the articulating cup.

Thirdly, The fractured end of the shaft of the humerus was touching the under edge of the articulating cup, and lying in juxta-position to the head of the humerus, but at an obtuse angle with it.

Fourthly, A line was running visibly between the top of the shaft of the bone and its head, with a perceptible depression between the two separated portions of bone, shewing the nature of the injury unequivocally.

Fifthly, Coagulable lymph had been thrown out around the injury, but was in progress of absorption.

Sixthly, The belly of the biceps muscle was attenuated, the muscle itself being disabled. It was this disability of the biceps muscle which was the cause of the inability in the movements of the fore-arm.

There was no union between the fractured head and shaft of the bone, and from the limited play of the humerus on the lower edge of the glenoid cavity, a false joint seemed in process of formation.

On Jan. 23d, 1840, three months after the injury, the patient died.

*Dissection of the Limb.*—Beneath the deltoid, the humerus close to the neck was found to have been broken into six pieces, and united by new bone. The glenoid cavity was seen empty, and covered with its cartilage; the axis of the limb being directed towards it. The head of the humerus was felt beneath the glenoid cavity, resting on the inferior costa, just below the cervix scapulæ, with its articulating surface directed downwards. It was closely invested by its capsular ligament, which was entire; the breach caused by the dislocation having been repaired. On opening it, the head of the bone presented its usual appearance, retaining its cartilage, and being smooth and polished. The tendons of the spinati and subscapularis appeared thickened; but were entire, as if they had been torn and repaired. The long tendon of the biceps was torn from its origin, and entangled among the fragments of the fracture, above which it could not be traced.

The motion enjoyed by the articulation was very limited, being restrained by a process of union going on between the glenoid cavity and a fragment of the humerus lying in contact with it. This union was chiefly by means of an imperfectly ossified matter, and therefore allowing a slight degree of motion. This union might probably have been prevented by a continuance of passive motion.

Cases and Observations illustrative of Renal Disease accompanied with the Secretion of Albuminous Urine. Memoir the Second. By R. Bright, M.D. F.R.S. Physician Extraordinary to the Queen.

The main object of Dr. Bright in writing the present paper is to correct a misconception of which he complains—namely, that he maintains the occurrence of albuminous urine to be always and necessarily connected with that organic disease which, in its various shapes and modifications, has been so fully described. "Now the truth is," he adds, "that I have never written upon the subject without studiously stating the contrary opinion, and declaring that I considered the disease, in its commencement, entirely functional." He quotes passages in corroboration of this statement from his first publication in 1827, from his second in 1831, and from a third memoir in 1836.

"After these quotations, I trust I shall be exonerated from the imputation of asserting, that albuminous urine cannot exist without disorganization having taken place in the kidney. At the same time, it must be confessed, that within a very limited time after the occurrence of the morbid condition of the urine the disease has generally proceeded so far as to bid defiance to the best-concerted measures for its removal, and has probably begun to produce a structural change in the organ."

In the present communication, Dr. Bright has brought forward a few cases, which, he thinks, may assist us in forming a fair estimate of the degree of success which we may reasonably expect to attain in the treatment of albuminous urine; sometimes effecting complete cures; sometimes diminishing the morbid condition of the secretion; but more frequently only rendering the various symptoms less formidable, and the fatal result less immediate. For, no one, he believes he may say, has it in his power to adduce a long series of perfectly successful cases, though relief is frequently afforded, and results produced such as many have considered cures. Although few of them, says Dr. Bright, deserve that designation, they are calculated to afford some comfort and encouragement while we are engaged in the hitherto discouraging search after efficient remedial agents when the disease has assumed its confirmed form, and to excite a hope of being useful amidst the still more distressing disappointments to which we must submit when the disease has become organic.

Dr. Bright details, with considerable circumstantiality, twenty-four cases. These occupy some fifty pages of the "Reports." It would be useless on our part going through each, but their general tenor, the facts they prove, and the conclusions that they point to, may, in some measure, be gleaned from their respective headings, a kind of abstract of their characters. These we shall cite, and then notice two or three more fully.

Case 1.—Exposure to Cold—Albuminous Urine—Anasarca—strict confinement to bed—diaphoretics—elaterium—permanent cure.

Case 2.—Albuminous Urine—Ascites and Anasarca—warm bath—purging—mineral acids—complete cure.

Case 3.—Albuminous Urine—Anasarca—confinement to bed—simple salines—all symptoms removed.

Case 4.—Albuminous Urine—treated by compound ipecacuanha powder, &c. &c.—great improvement during many years—urine still coagulable.

Case 5.—Anasarca after Scarlatina—urine albuminous—complete cure by confinement to bed.

Case 6.—Albuminous Urine after Scarlatina—strict confinement to bed.

Case 7.—Albuminous Urine—Anasarca—bleeding—gentle purging—light tonics.

Case 8.—Albuminous Urine after Scarlatina—excessive anasarca—death—mottled kidney.

CASE 9.—Albuminous Urine, recent Case—strict confinement in bed—purgatives and antimonial diaphoretics—anasarsa cured—urine still albuminous.

CASE 10.—Albuminous Urine, a recent Case—strict confinement to bed—antimonials—anasarca cured—urine still slightly albuminous.

CASE 11.—Albuminous Urine—general anasarca—bleeding—elaterium—anasarca cured—urine remained very slightly albuminous.

CASE 12.—Albuminous Urine—confinement to bed—bleeding—elaterium—great relief—urine still slightly albuminous.

CASE 13.—Albuminous Urine—bleeding—much relief from gentle diuretics and purgatives—urine still slightly albuminous.

CASE 14.—Albuminous Urine—anasarca—infus. digitalis, &c. &c.—anasarca cured—urine still coagulable.

CASE 15.—Albuminous Urine—elaterium—great improvement.

CASE 16.—Albuminous Urine—elaterium—great relief.

CASE 17.

CASE 18.—Albuminous Urine—severe cramps—serous effusion.

CASE 19.—Albuminous Urine during at least two or three years—habitual cramps—death, with violent spasm of the muscles—urea in the serous fluids.

CASE 20.—Albuminous Urine—severe epileptic fits—death.

CASE 21.—Albuminous Urine—very defective vision—spasm—coma, with death.

CASE 22.—Albuminous Urine, probably of several years' duration—very defective vision—inflammation of pericardium from exposure—convulsion—death.

CASE 23.—Albuminous Urine—imperfect vision, and other cerebral symptoms—serous inflammation—phosphatic deposits in one kidney.

CASE 24.—Albuminous Urine in a case of diabetes mellitus.

The titles of the preceding cases furnish a sufficient notion of their general character, and present the practitioner with an idea of what he may commonly expect in the treatment of albuminous urine. We shall now take one or two of the more striking, and especially of those which seem to illustrate the beneficial effects of remedies.

We shall begin with,—

CASE 1.—*Exposure to Cold—Albuminous Urine—Anasarca—Strict confinement to Bed—Diaphoretics—Elaterium—Permanent cure.*—Feb. 5, 1837. Dr. Bright saw a gentleman, aged 35, of good constitution, who, eight days previously, the weather being very cold, came from Maidstone on the outside of a coach; and on the same evening his wife observed that his face was swollen, and he complained of some pain in the loins. The swelling has since continued and increased, shewing itself in various parts of his body. His skin was dry, except the palms of the hands and soles of the feet; the urine scanty, with lateritious sediment: on the application of heat, it first became clear, and then coagulated freely. A third of a grain of elaterium, given the day before, had somewhat reduced the anasarca.

He was ordered to remain strictly confined to bed, in a warm room; to have a light milk diet; and to take a pill composed of three grains of James's powder and two of extract of conium, three times a day; and a draught, containing three drachms of the liquor. ammon. acet. He was also to repeat the elaterium about once a week.

Next day he had a profuse perspiration, which was succeeded by a permanent moisture of the skin. On the 8th, pulse 80, full: urine, two quarts, of a high brandy colour, and slightly dingy, but free from lateritious deposit: still decidedly coagulable by heat, but in a less degree than before. He was allowed a little vegetables and a small quantity of fish. To take the third of a grain of elaterium the next morning, and continue his other medicines.

Feb. 20. The perspiration has been almost constant, and he feels weakened by it. For the last few days, the urine has deposited a slight brown sediment, apparently from the presence of some of the red particles of the blood. To leave off the James's powder, but continue the other medicines.

March 6. Up to this time, no alteration has been made in the plan of treatment; except that a little sulphate of magnesia has been added to his draught, and the James's powder has been resumed. He feels and looks quite well; but the urine is too abundant, and he is called upon two or three times each night to pass it. It looks pale and watery, and has a slight dingy appearance; but is not the least coagulable by heat. The perspiration is now rather deficient. Twenty minims of the liquor. antim. tart.: to be added to each dose of the mixture, and the pill to be continued.

March 13. In appearance, he is now quite well, though the urine has still a slight dinginess. The quantity is natural, and it is not the least coagulable. Skin perspirable.

Dr. B. now took his leave. The gentleman has remained quite well since.

The *Second Case* was that of a man, aged 56, a coal-whipper, who had always enjoyed good health and equally enjoyed both gin and porter. The anasarca and ascites had begun eleven days before his admission. The treatment consisted of active purging, which diminished the ascites, but not the anasarca—hot baths which made him sweat, as we may presume only coal-whippers can, for the perspiration is described as "running through the *bed,* and standing in *puddles* on the floor,"—and, lastly, infusion of gentian with the hydrochloric acid. Dr. Bright remarks on the case:—"if it were allowable to speak of our mode of curing the disease from the success of a single case, we should be induced to say, act freely on the skin, lower action by purging, then improve the tone of the kidney by the mineral acid, and the cure is complete: and though I am not, as I have said, sanguine of great success, yet these appear most rational indications, and are, I believe, capable of being frequently carried into effect, if the patient will submit to confinement to bed for many weeks, during the action of remedies."

The next *case,* to which we shall turn is the *fourth,* because it is, we think, a sample of a class far from uncommon.

Case 4.—*Albuminous Urine—treated by Compound Ipecacuanha Powder, &c. &c.—Great Improvement during many years—Urine still coagulable.*—"March 9, 1835. A man, aged about 25, pale and scrofulous in appearance, and deeply pitted with the small-pox, came to me, labouring under anasarca, and having albuminous urine. His illness commenced, as far as he knew, eight months before, with a diarrhœa, which lasted three weeks; and, when getting better, he went into the country for a month; but there his legs began to swell, and anasarca proceeded up the thighs and abdomen. He had been taking various remedies for the last four or five months, without deriving material benefit. I found the urine of a pale straw-colour, exceedingly coagulable by heat and by nitric acid: it became frothy on agitation, and remained so. The whole quantity he passed was very considerable, and he suffered much from irritation of the bladder, so that he was always obliged to rise three or four times during the night."

Extracti Humuli. Extracti Uvæ Ursi āā gr. xv. contunde et in pilulas decem divide, e quibus sumat unam ter quotidie.

Milk diet. Flannel.

On the 23rd, the urine less coagulable—only one call at night—cough, and relaxed bowels.

℞. Sodæ Subcarbonat. ʒij. Pulv. Uvæ Ursi ʒij. Pulv. Ipecac. Comp. ʒss. in pulveres xij. divide, e quibus sumat unum ter quotidie.

Habeat Mistura m Cretæ aromat. pro re natâ.

On the 17th of April, the skin being dry, he was ordered 4 *grs. of Dover's*

*powder twice or thrice daily,* which was soon increased to five grains thrice daily.

In June, he had improved, but a slight periosteal enlargement had appeared on his left shin; he had taken much mercury. The iodide of potassium in infusion of cascarilla was added to the Dover's powder. On the 9th July, he was ordered—

Pulv. Ipecac. Comp. ʒj. Soda Carbonat. ʒij. Pulv. Uvæ Ursi ʒij. In pulv. duodecim divide, e quibus sumat unum omni nocte.

Infus. Gentian. C. ℥v. Potassæ Hydriodat. ʒiss. Tinct. Aurant. ʒiij. Tinct. Card. Comp. ʒiij. Syrup. Aurant. ʒij. Sumat cochl. mag. i. ex cyatho aquæ semel vel bis quotidie.

Passing over some intermediate reports we may cite the concluding ones.

"Oct. 30, 1839. Nearly four years had elapsed since I last saw this patient; and he now came to me, telling me that he had continued in good health till within the last two or three months; but when he had occasionally felt some of his old symptoms, he had recurred to the mixture. He had passed his time in the comfortable discharge of his business; had experienced no swelling; his skin natural, and his urine not more frequent nor in larger quantities than usual: at present, however, he suffered from frequent calls to pass it after going to bed; and on rising in the morning, he had headache, with frequent nausea, proceeding often to vomiting. I had no opportunity of seeing his urine at that time. —I ordered an effervescing draught occasionally.

Nov. 3. I examined his urine; it was not acid, and became very faintly opaque by heat; it coagulated freely with nitric acid: specific gravity 1012. He passed a large quantity of urine at night, but little in the day: his ankles had swollen slightly of late."

We give this case, as we before observed, because it may be loosely said to represent a class. It is by no means rare to see persons go on for year after year with albumen in the urine, more or less irritability of the bladder, a tendency to anasarca, and indifferent, rather than positively bad health. We fancy that it is the quantum of disease in the kidney, rather than the influence of particular medicines, that enables the patient to creep on thus little better or worse for an almost indefinite time. We have, at all events, seen this occur under very different remedies, and regimen and the avoidance of the causes of disease have, perhaps, had as much to say to it as physic.

*Albuminous Urine in connexion with Scarlatina.*

Dr. Bright remarks:—

"During the autumn and winter which have just passed (1839-40), we have experienced an almost epidemic prevalence, in London and its neighbourhood, of that anasarca with albuminous urine, which has been long known to accompany or follow scarlatina. The attack of the original disease has often been slight; the rash sometimes scarcely perceived; the sore-throat often mild; and the affection of the urine has frequently shewn itself while the rash or sore-throat has still existed. Hæmaturia has occasionally occurred during the severity of the attack; but more commonly this symptom, with or without anasarca, has followed on the subsidence of the fever, or during the early convalescence. The number who have offered themselves as out-patients, or have been admitted into the wards of Guy's Hospital, under such circumstances, as well as the cases which have occurred in private practice in other parts of London, have been quite unprecedented, within the limits of my experience.

This I consider one very important form of the disease, and one which very generally admits of cure; but I have seen and heard of several fatal cases, and have had the opportunity afforded me of examining the kidneys of some who have died. In some instances, there has been obvious irregular congestion, without apparent lesion of structure; but in one or two, where there was reason to believe that a foundation had been previously laid, the more usual appearances of advanced structural derangement have been ascertained.

With regard to the occurrence of albuminous urine after scarlatina, I have already said, in a former paper in these Reports,* that I consider it as often laying the foundation for the future disease, or as an evidence of the strong tendency existing in the constitution, even when the cure has been apparently complete."

Four cases are detailed. The three first terminated favourably, and as there was nothing particular in the treatment, nor indeed in the circumstances, we may dismiss them. The fourth, however, has pathological interest at all events.

Case.—*Albuminous Urine after Scarlatina—Excessive Anasarca—Death—Mottled Kidney.*—John Wiseman, aged 16, admitted November 16, 1839. He has always enjoyed a good state of health till about a month ago, when an eruption of scarlet-fever made its appearance on the body. During the attack he continued to be engaged in the discharge of his duty, frequently exposing himself to damp and cold by going into the cellar. A medical gentleman, who attended, said he had scarlatina. A fortnight ago, he perceived a swelling about the eyelids, which increased consideraly towards evening, attended by pain and giddiness in the head: the next morning, the swelling had extended to the face; and in three days more, to the upper and lower extremities, scrotum, penis, and abdomen. At this time he complained of sickness and trembling of the limbs, accompanied by coldness of the feet· his urine was small in quantity.

*Present Symptoms.*—Has a general œdema over the body, with great tension of the skin, more strikingly marked in the abdomen, scrotum, eyelids, face, and lower extremities: great thirst: pain in the loins on pressure: skin dry and hot: tongue furred and injected: respiration hurried and laborious: urine of a dingy orange colour, small in quantity, very coagulable by heat and nitric acid. He was ordered to be freely purged by elaterium and supertartrate of potash; but this produced great sickness, without better action on the bowels.

*Nov.* 17. Has had no sleep: constant vomiting: pulse 114, small and sharp: skin dry: respiration hurried and laborious: tongue somewhat brown: pain in the head: has passed six ounces of urine, the same in character: great thirst, and pain in the loins: cannot lie down.

Baln. tepid.

Hydr. c̄ Cret. gr. v. t. d.

Acid. Hydrogen. ex M. Mag. t. d.

18. Has passed a very restless night: pain in the loins undiminished: great pain and tension in the scrotum: the skin burning: œdema remains the same: urine very scanty, and coagulable.

Incidatur scrotum.
V.S. ad ℥x.
Fot. Papav. lumbis.

Only ℥iv. of blood could be obtained; which was buffed, and of a light colour.

19. Died.

Sectio Cadaveris.—The whole body much swollen, and anasarcous. The right ventricle contained a small quantity of fluid.

*Chest.*—The right pleura contained about a pint of clear serum; the left about eight ounces of dark-coloured serum. The lungs were only compressed from fluid. The cavity of the pericardium contained three ounces of straw-coloured

* Vol. I. p, 339.

serum, mixed with a few flocculi of albumen. There were evidences of inflammation of the peritoneum. The liver was congested.

Kidneys of their natural size: tunics adherent; the cortical portion much mottled with white deposit, so that everywhere it appeared of a whitish colour; the tubular portions natural. The other viscera healthy.

Dr. Bright rather regrets that he did not bleed earlier. We lately saw a case which was rather interesting. A young gentleman was observed by his friends to grow languid and rather puffy about the face and ankles. Yet they thought little of the circumstance, until the swelling of the face had increased, and the boy was evidently ill. Six weeks had passed from the first manifestation of anasarca when we were consulted respecting him. The anasarca was general, and, in the lower limbs rather considerable—there was ascites with some tension of the belly—some dyspnœa, without evidence of thoracic implication—and scanty urine, loaded with the lithates, and coagulable. The child was of a delicate and scrofulous habit, had always been ailing, and had so little pulse that we hesitated to bleed. There was slight tenderness of the abdomen, but it seemed due to the tension of the skin, for equal tenderness was felt in the nates, and the lower limbs. We ordered leeches to the belly, and salines with the infusion of digitalis and purgatives. But the child rapidly grew worse, the anasarca increased, coma set in, and in three or four days he died. We supposed that the kidney was affected, as the symptoms rather pointed to that than to any other organ of consequence. The fact, however, was otherwise. For, on dissection, the kidneys were sound, no congestion, even, of consequence existing in any part of them. The thoracic organs were sound also. The disease consisted in inflammation of the peritoneum, in the cavity of which was a good quantity of turbid serum, with flakes of lymph, and even purulent deposit in dependent parts or corners, such as the pelvis, interstices between the intestinal convolutions, &c. We certainly *did* anticipate increased vascular fulness about the kidneys, and we *did not* anticipate so *much* inflammatory action about the peritoneum. We fancy, if the truth were told, that there are few persons in any practice, whether public or private, who have not occasionally found more inflammation of a serous membrane, after death, than they had bargained for during life. Such inflammations not unfrequently come on slowly and insidiously, both in the chest and the abdomen.

To revert to albuminous urine, in connexion with scarlatina, Dr. Bright remarks:—

"Mr. Streeter kindly permitted me to see the kidneys of a child who died this winter under the form of disease of which we now speak, but in which no permanent change had taken place: the evidences of congestion, and more particularly in the tubular portion of the kidney, were most marked.

In a case of a child seven years old, which Mr. F. Toulmin had an opportunity of examining, one of the kidneys was mottled, and the capsule adherent."

Two cases are detailed with the view of exhibiting the effects of antimonials. We shall select one.

CASE 10.—*Albuminous Urine, a recent case—Strict confinement to Bed—Antimonials—Anasarca cured—Urine still slightly Albuminous.*—William Arnold, aged 16, was admitted, under my care, into Guy's Hospital, Sept. 2, 1835, with general anasarca. A fortnight ago, during the very hot weather which then prevailed, he had been exposed to some rapid alternations of temperature; and on awaking one morning, found his face swollen: shortly after, his ancles and other parts of his body began to swell. He experienced aching pain in his loins; and his urine became red or brandy-coloured, and was deficient in quantity.

At the time of his admission, pulse 84, urine dingy and turbid, sufficient in quantity, but very coagulable by heat, forming a complete curd-like mass.

I ordered him to go to bed, and remain there; and gave him the liquor. ammon. acetatis, with solution of tartarized antimony, every fourth hour.—Milk diet.

The following day he was cupped from the loins, to twelve ounces; and took a brisk purgative.

*Sept.* 4. The skin continues dry; face more swollen; pulse 84; lumbar pain relieved by the cupping. He speaks of a palpitation of the heart, to which he has been occasionally subject all his life, but which has increased within the last few days.

Extract. hyoscyami gr. iij. Antimon. Tartariz. gr. $\frac{1}{8}$, fiat pilula ter quotidie sumenda."

He improved, and, on the 18th, the antimony was increased to the 4th of a grain every six hours. Giddiness occurring, the henbane was omitted on the 22d of October. On the 7th of November, he is described as feeling himself in perfect health. He was ordered to take five grains of subcarbonate of ammonia three times a day, and leave off his other medicine.

He continued in the hospital till the 23d; when he felt so well, that he chose to return to his occupation. His urine still shewed very slight signs of albumen. He had experienced no return of the anasarca, though he had been walking about the ward, and had frequently been down stairs for the last fortnight: his pulse, however, was still very irritable.

Two cases are given for the purpose, apparently, of shewing the effects of bleeding and elaterium. We shall cite one.

CASE 11.—*Albuminous Urine—General Anasarca—Bleeding—Elaterium—Anasarca cured—Urine remained very slightly Albuminous.*—David Neville, middle-aged, a river-side porter, accustomed to drink, admitted April 5. A fortnight ago he was quite well, except a slight cough: about that time general anasarca came on, first in his legs and thighs, then in his face: his urine is very scanty, less than half-a-pint in twelve hours; dingy; coagulable: pulse 84, labouring: he complains of some pain in the loins: no head-ache. He was bled immediately to fourteen ounces, and had his bowels opened: the quantity of urine gradually increased, and became abundant in ten days' time; but he remained in the hospital till the 4th of June, subject to relapses of the swelling; and when he left the house, his urine, though less coagulable, still became opaque by heat.—The treatment adopted was chiefly bleeding and purging; he was bled three or four times, and cupped, and the blood was buffy; and the elaterium, given in doses of one-eighth of a grain till it purged, was the most effectual purgative; he also took some diuretics at different times, and thought the decoction of pyrola the best.

The *fifteenth* and *sixteenth* cases are offered as samples of treatment by elaterium.

The *sixteenth case* is a pretty good example of the good effects produced by it.

CASE 16.—*Albuminous Urine—Elaterium—Great Relief.*—C. Hellyer, aged 40, admitted Sept. 25, 1839, a man of a pale and leucophlegmatic appearance, whose occupation was that of a leather-dresser, being constantly exposed to wet and cold. His history is the following:—twenty years ago, had a slight attack of dropsy in the face and head: was cured by cupping. Ten years ago, had another attack, more severe than the former. Three years and a half ago, had another attack, which lasted for six weeks. Five weeks ago the present illness commenced, for which he has taken medicine ineffectually. His skin had always been dry.

At present, the scrotum, thighs, legs, and all depending parts, are much swollen, from anasarca: the eyelids are also swollen: skin hot and dry: urine very scanty, of a dingy colour, and decidedly coagulable by heat: he is also called upon in the night four or five times to pass it: he states that in health he makes very little urine, and has frequently remarked its dingy appearance. He was ordered the following medicine:

Julep Ammon. Acet. c̄ Vin. Antim. t. d.
P. Guaiaci gr. v. Pulv. Jacobi gr. ij. t. d.

and ordered to keep his bed.

Sept. 26. Pulse much better: states that he has passed a quart of urine in the night, of the same character.

30. Pulv. Elat. Comp. gr. xv. st.; et. rep. cras mane, si opus fuerit.

Oct. 2. His bowels were opened twelve times by the elaterium: stools watery and copious: he feels greatly relieved by its action: the swelling in the scrotum and face is greatly diminished, and the skin feels more perspirable: urine the same in every respect. The elaterium was repeated on the 8th, 12th, 16th, and 22nd of October. On the 20th, the report states that the swelling has disappeared from all parts: skin soft and perspirable: urine of a dingy colour: called upon in the night five times to pass it, and in large quantities; coagulable by heat. On the 26th, he felt so well, that, contrary to advice, he left the hospital. Every time he took the elaterium, he experienced the most marked benefit.

The *fourteenth case* was treated by infusion of digitalis, to shew the effects of which we shall cite it.

CASE 14.—*Albuminous Urine—Anasarca—Infus. Digitalis, &c. &c.—Anasarca cured—Urine still coagulable.*—Drusilla Wilson, aged 56, admitted, December 11, 1830. She stated that her present illness began two months ago, with loss of flesh and strength; and about a month ago her lips began to swell. At the time of her admission, her legs were very much distended with œdema. Pulse 120, sharp, with some degree of jerk; bowels open: urine scanty and coagulable.

Infus. Digital. ℥i. ex Misturâ Camphoræ, ter die.

*Dec.* 12. Urine about 1½ pint, which she thought a considerable increase: pulse 100, and more natural.

Rep. Medicamenta.

On the 4th Dec. swelling nearly gone: urine above four pints in twenty-four hours, and not at all coagulable: pulse 88.

On the 7th, she was ordered steel wine thrice daily. At the latter end of the month she contracted a catarrh from the severe weather, which seems to have thrown her back, for we find, on the 8th Feb. urine still decidedly coagulable; but she says she feels quite well, except an occasional pain in the loins; and she wishes to return to her family. We should state that the uva ursi and soda were tried, apparently with disadvantage.

Dr. Bright sums up the first seventeen cases with these brief remarks:—

"Similar instances might be multiplied to a very great extent; but they would still only serve to shew, that the most recent cases are often capable of cure by depletion, either by bleeding or purging, according to the acuteness of the attack, and by promoting gentle diaphoresis both by internal remedies and by strict confinement to bed;—and that many other cases admit of being so much relieved, that the patient is himself often confident of having obtained a perfect cure; being so free from all obvious symptoms of disease, that it requires the experience and knowledge of a physician to discover the latent malady, which however unfortunately, is soon brought to light by the necessary exposure of active life."

The last *seven cases* are detailed with the view of exhibiting some of the complications or results of renal disease. We shall select the more striking only.

The *twenty-third case* offers some points of interest.

Case 23.—*Albuminous Urine—Imperfect Vision, and other Cerebral Symptoms—Serous Inflammation—Phosphatic Deposits in one Kidney.*—Dr. B. was first consulted in the case of Mr. B., Nov. 15, 1837, a fine-looking man, approaching fifty years of age, who had served in the army many years, both in tropical climates and in the Low Countries, and had suffered twice from attacks of the yellow fever in the West Indies. In general, however, he had enjoyed very good health, till the last two years; during which time he had been accustomed to wake in the morning with a severe headache, with a most painful throbbing and beating in the eye-balls, which has been generally followed by vomiting, the moment he has risen from bed. These symptoms at first shewed themselves about once in a month; but have increased so much in frequency of late, that they often occur three times in the week; and occasionally sickness will come on suddenly in the day: and for the last few months there has been almost constantly a dimness in the right eye, so that if he attempts to read with that eye the letters are interrupted. He has no pain anywhere, except a little in the right kidney, which, however, is not tender on pressure; and he speaks of a little occasional pain in the nape of the neck, or near the insertion of the muscles, on the right side of the occiput.

He is now always disturbed in the night by very frequent calls to pass water, which is abundant, and very limpid: in the day-time he is seldom called upon for that purpose above twice, and the secretion is natural in appearance. Dr. B. found the pulse rather irregular, and detected a singular squeaking and croaking with the first sound of the heart. Supposing that there was a threatening of an apoplectic attack dependent, probably, on diseased arteries, Dr. B. ordered appropriate remedies, with temporary benefit; but, in December, Dr. B. was informed that the sight was so much impaired as to preclude reading.

"*Jan.* 27, 1838, I saw him again: he had lost flesh: his complexion was sallow: he had been suffering from dyspnœa for the last week, with pain, first at the lower part of the chest in the left side, and then in the right side, near the mamma; and, on examination, I found a *frottement,* from recent pleuritis, beneath one scapula.

Some of his symptoms had of late greatly improved, and his morning sickness was quite gone; but his loss of sight was so great, that he could neither read nor write. He still stated, that the frequent calls to pass urine during the night were very distressing, though there was nothing of the kind in the day; and the vessel was filled during the night with pale-coloured urine. He had observed this, in some degree, nearly three years, but it was much worse for some months. This led me to examine the urine; which I found highly albuminous, forming the usual precipitate, both with heat and nitric acid; and I now, for the first time, became acquainted with the real nature of the case. I had no doubt that the derangement of the kidneys had existed for two or three years; that they had in all probability already passed into a state of hopeless disorganization; that the cerebral affection had depended in a great degree upon the state of the kidneys; and that during the last week he had been suffering from an attack of pleuritis. In this case there had at no time been any signs of anasarca; and the skin, though rather dry, was capable of being brought into a state of perspiration by exercise.—The means made use of speedily put a stop to the recent inflammatory symptoms; but those which were referable to the kidney continued with little abatement; indeed, the calls were frequently not fewer than ten in the night; and a trial was made, by his medical adviser in the country, to control this symptom, by adopting an animal diet; which was to a certain degree successful: but the sickness of the stomach recurred; and in the beginning of April, when he had returned, after an absence, to his usual residence, he was seized with a sudden and alarming increase of symptoms,—dyspnœa coming on, and increasing rapidly, the slightest movement producing the greatest distress, with utter inability to lie down; œdema of the lower extremities, hands and face, pulse seldom under 120."

Under the use of abdominal fomentations, affection of the mouth by mercury, and the pyrola umbellata he rallied marvellously, though only for a season. About the middle of 1838, he grew worse, the dyspnœa became excessive, the anasarca increased so as to require punctures, dysentery set in, and, the night before his death, he quite lost the use of his right side.

*Dissection.*—Body much emaciated; with very little appearance of œdema, except in the right arm.

Right cavity of the chest filled with whey-like fluid, not less than three pints, compressing the lung into one-fourth its natural size. The whole costal pleura, and the diaphragm and part of the lung, covered with a thin coating of lymph, alternating with spots and patches of vascularity and ecchymosis: some bridles of firm lymph from the lower lobe of the lung. The upper lobe, where not compressed, was œdematous.

The left cavity contained a few ounces only of straw-coloured serum. Pleura healthy, except some strong old adhesions.

*Heart,*—Three times the natural size: the left ventricle particularly enlarged by eccentric hypertropy. The aortic valves quite healthy. The mitral valves, except one or two spots of thickening, natural. The right side of the heart rather hypertrophied. One ounce of fluid in the pericardium. Aorta enlarged, but not diseased.

*Liver* rather large and pale; held up to the diaphragm by very old long adhesions. The gall-bladder filled with small irregular angular calculi, in number 97; and one of a rather larger size near the opening of the duct: ducts pervious: stomach, spleen, and pancreas, healthy.

*Kidneys.*—The right, very small, and completely filled with a white paste, which, on examination, was found to consist of the earthy phosphates; the cortical portion forming a mere bag: the ureter of that side large and thick. The tunics adhered quite firmly to that kidney.—The left kidney rather larger than natural, soft and tough in texture, completely mottled throughout, and granulated on its surface, affording a very well-marked specimen of the disease. There were several small vesicles on the surface. The tunics did not adhere, but tore off easily, leaving a rough surface.—Bladder healthy.

*Head.*—Dura mater pale, and scarcely shewing any vessels. Arachnoid, and pia mater, pale: a very little serum in some of the sulci. The arachnoid separated easily. The brain was throughout exsanguine; but the only disease was a small softening about the size of a french-bean, apparently once an apoplectic clot, in the centre, rather posteriorly to the left hemisphere, hardly so low as the roof of the ventricle. No serum in the ventricles, and none in the base. The spinal cord was examined, but no disease discovered.

Dr. Bright remarks:—

"This case presents some points of interest. The severe headaches, and the interrupted vision, were marked symptoms when first discerned, though this was probably very long after the commencement of the disease. The flattering prospects, sometimes held out—the affection of the heart, probably a sequel to the renal disease—and the extensive serous inflammation which at length attacked him, and proved fatal—are all characteristic."

None can be more disposed to attach importance to renal disease than ourselves, yet we confess that we doubt the strict justice of referring to *it* as the primum mobile in cases like the preceding. The heart we conceive, has as much right or more to the distinction—and, whether we look at the history, the symptoms, or the amount of lesion, it would be difficult to postpone the claims of cardiac disease to those of renal. Not that we mean to deny the tendency created by the latter to serious secondary alterations; far from it. We only mean to limit the application of views drawn from that tendency, and to enjoin caution in their adoption.

"There is another point of interest connected with the case I have last detailed. I refer to the collection of phosphatic deposit discovered filling the kidney; the remaining parts of which, as well as the kidney on the opposite side, had all the characters of advanced granulation; thus affording a striking example of the co-existence of that deranged action by which the phosphates are deposited, and that by which the albumen is secreted. We have thus an additional proof, if any were wanting, that the albuminous action admits of various complications; a fact which, indeed, has already appeared, from the details of many cases which have at various times been stated: for it is notorious, that the urine, which is generally acid in this disease, sometimes becomes alkaline, without losing its albuminous character; although the albumen may be in some degree modified: still, the lithic deposits are those which usually accompany it: and when calculi have been passed during the existence of the albuminous tendency, I have frequently found them to be composed of lithic acid. In the present case, however, we have the phosphatic deposit in large quantity, and in very tangible form.

It would be in vain to speculate on problems so imperfectly worked out; but, from the knowledge we possess of the co-existence of various forms of disease in the kidney, we might be inclined to suppose that no diseased impregnation of the urine is so completely dependent on what may be styled the organic agency of the kidney itself, as albumen. In diabetes, and in the different diatheses which produce calculous concretions, it is probable that the blood, as prepared by the various processes of the body, is much more in fault than the kidney; and it is truly extraordinary to what an extent, and to what duration, these diseases may proceed, without the kidney betraying any change; whereas it is certain that the ultimate tendency of the albuminous action is to disorganize the structure of the kidney: and it is still more extraordinary to find, that when the kidney is actually disorganized, it is still capable of undergoing that functional change which attends diabetes itself; or, at all events, that the existence of the albuminous arrangement does not prevent the existence of diabetes, and the secretion of saccharine matter. In confirmation of this, I will state a case which has lately come to my knowledge, of the well-marked combination of albuminous urine with diabetes mellitus, in which Dr. Bostock had an opportunity of verifying the fact, by chemical analysis."

This case we shall notice.

Case 24.—*Albuminous Urine in a case of Diabetes Mellitus.*—William Schooling, aged 33, admitted Feb. 6, 1840, is a single man, a coachman; of intemperate habits; has never enjoyed good health. About a year and a half since he had a feverish attack, with excessive thirst: his urine at that time was natural, both as to quantity and appearance. Since that time his health has gradually declined, and he has become greatly emaciated.

On his admission, he complained of great weakness, pain in the back and loins; on the right side, increased by pressure: great thirst, and dryness of the mouth: gums red and spongy: skin dry: appetite craving. He sleeps well; but is forced to rise three or four times during the night, to pass water—quantity, in twenty-four hours, four quarts: sp. gr. 1034. The peculiar odour of hay is perceptible in the breath: pulse 82, sharp, but compressible: tongue slightly furred: bowels confined.

Animal food and creosote were prescribed. But he grew worse. In the night of the 23rd, his urine began to pass involuntarily, and on the 24th, the report states, that, he now lies in a comatose state: extremities cold: the hands firmly contracted: the mouth drawn to the right side: the pupils contract to the stimulus of light: pulse scarcely perceptible. He died at 6 p. m. of the 25th.

*Dissection.*—The vessels of the brain contained but little blood. There was a large quantity of fluid, both in the cavity of the arachnoid, and between it and the pia mater; but no unusual quantity in the ventricles.

The substance of the brain was throughout healthy.

There were two drachms of fluid in the pericardium, and traces of former pericarditis.

The heart was of natural dimensions, but pale, fat, and flabby. Its auriculo-ventricular valves slightly opaque.

There were old adhesions on the whole of the left lung, but no fluid in either pleural cavity.

The right lung was healthy. A portion of the apex of the left was hepatized in the first degree.

The abdomen contained four ounces of clear serous fluid, and there was slight adhesions of the omentum to its walls. The viscera generally were pale; the stomach distended; the intestines contracted: all were, in structure, healthy. The liver alone was rather fuller of blood than natural. The kidneys were of their usual size, flattened anteriorly: they were pale, and slightly mottled, but of natural consistence, and not granulated on their surface. The pelvis of each was rather more vascular than usual.

Dr. Bostock, on analysis, found both sugar *and* albumen in the urine. Dr. Bright winds up with a few hints on remedies.

"In the first steps, and the more acute forms of disease, bleeding may be considered the most important remedy: but this is, of itself, wholly inadequate to cure, unless we purge freely, and at the same time call upon the skin to do its duty. Of all the measures for effecting this latter purpose, the strictest confinement to bed is the most effectual; and without that, I do not believe that, in this climate, we have a chance of cure. That preliminary, however, being adopted, antimonials are probably the best diaphoretics: but the liquor ammoniæ acetatis is likewise very useful: and a simple saline draught of citrate of potash or soda, is, I believe, when diligently persisted in, of much avail: and the warm bath, in its various forms, may in many cases be brought to act most beneficially.

Amongst the purgatives, I shall only mention, that elaterium and jalap, with the bitartrate of potash, appear to me the most effectual. When the disease has made further progress, and has become chronic, perhaps organic, I should still recommend the greatest attention to the full effects of purgation, and to the state of the skin, and to protection from atmospheric changes; and I am more and more impressed with the probability, that if a complete change of climate were tried, great benefit might result. A voyage to the West Indies, and a residence in one of the more healthy islands, often produce a great change in the constitution, acting chiefly upon the pores of the skin. We have, at least, the negative experience, that confirmed cases rarely recover in this country, whatever treatment be adopted; and the skin being always more or less inactive, suggests most forcibly a change of climate as likely to promote its function. It is the doubt, and uncertainty with which this disease is often viewed, that interferes with our recommending this bold measure, or, if recommended, interferes with its adoption: and I trust that the perusal of a few such cases as I have brought forward on this occasion, will assist in producing a conviction of the actual existence of this disease, and of such an approach to incurability by any means we at present possess, that a physician should feel no more compunction in recommending the expatriation of his patient with albuminous urine, than he would in a case of incipient or threatened phthisis.

There are certain remedies, whose actions in this disease are less obvious than those to which I have referred; but many of them probably act by affording a degree of stimulant or astringent action to the kidney: of these I may mention the mineral acids as applicable in the declining stages of more acute attacks; the uva ursi, in its different preparations, in the chronic disease, the pyrola umbellata, and the diosma crenata where great irritability of the urinary organs exists—a remedy which I have been led to adopt, in many cases, from the very favourable reports of Sir Benjamin Brodie: nor have I been disappointed of some good effect, though I should perhaps employ with greater confidence a long-continued course of soda, conium, and uva ursi. One thing, however, must be kept in mind, that whatever remedy is given to overcome a disease so chronic and confirmed, must be administered with exemplary patience and perseverance."

As regards those cases of renal disease and albuminous urine which are attended with irritability of the bladder, we must say that we have seen much more benefit from the diosma than from the uva ursi. Nor have alkalies or the alkaline subcarbonates corresponded with our expectations, or the recommendations of others; on the contrary, they have too frequently aggravated the frequency of micturition, and sometimes very materially. Sedatives have a certain influence upon the bladder, and, in some instances, the mineral acids conjoined with them are attended with a good effect. But, after all, candid men must admit that when once there is good reason to suspect the existence of organic lesion, a perfect cure is rare indeed.

The profession must be under deep obligations to Dr. Bright, for having drawn its attention to this serious malady. Our knowledge of its features has done much to give certainty to our diagnosis, and precision and rationality, if not success, to our treatment, in many cases formerly managed most empirically.

We shall introduce here as a pendent to Dr. Bright's paper, another by Dr. Barlow, headed thus:—

### Cases of Albuminous Urine, illustrative of the Efficacy of Tartar Emetic, in combination with other Antiphlogistic Remedies, in the Acute Forms of that Disease. By George H. Barlow, M.A. & L.M.

Dr. Barlow is anxious to contribute towards the remedying of this disease. His object was two-fold—to discover if the dropsy succeeding scarlatina depends on renal disease—and to treat it with the most success.

He relates two cases, with the view of proving the identity of the anasarca with albuminous urine which follows scarlatina, with the "morbus Brightii" from other causes. These cases, as they go to prove an important point in pathology, we shall quote.

Case.—*Scarlatina—Anasarca—Death—Kidneys diseased.*—Thomas Waters, aged 16, (Jan. 16, 1836), had a febrile attack about nine weeks ago, which was attended with sore-throat and great heat and redness of the skin, followed by desquamation, from which he in a great measure recovered; but a month ago he began to swell, first in his hands and face, and then over his whole body: for this he was attended by Mr. Howitt, of Walworth, under whose care he greatly improved, and about ten days ago he was nearly free from swelling; but about five days ago, it again returned, and has continued to increase up to the present time. He is now generally anasarcous; and there is a considerable quantity of fluid in the peritoneum. He has much pain in the loins, extending across the abdomen towards the umbilicus; and there is some tenderness, upon pressure, about the situation of the kidneys, especially of the right. Urine of a dingy-red colour, much loaded with albumen.

He was ordered small doses of tartar emetic, in combination with hyoscyamus; which he continued to use, with slight benefit, till the 3rd of February; when he was removed to Guy's Hospital, where he continued only five days: after which he was taken home at his own request, and died within two days.

The kidneys were very large; and the whole of the cortical structure of both was interspersed with a large proportion of pale yellow granular matter.

CASE.—*Scarlatina—Anasarca—Partial Recovery—Relapse—Death—Kidneys large and white.*—Elizabeth Dawkins, aged 11, (March 23, 1839). Has generally enjoyed good health. A month ago, she suffered from severe and well-marked scarlatina; from which she was slowly convalescent, when it was observed a few days ago, that her face was swollen. This swelling has continually increased to the present time; and she is now universally œdematous. She complains of headache, and pain across the chest, aggravated by inspiration. Tongue furred: bowels confined: pulse sharp: urine scanty, of a deep dingy-red, and very albuminous.

She was treated with purgatives; tartar emetic, with acetate of ammonia; and small doses of ipecacuanha. On the 26th, she was apparently so much better that she exposed herself, and on the 2nd April Dr. Barlow found her insensible, with well-marked symptoms of severe cerebral disease, and extensive anasarca. From this state she was recovered with some difficulty; and appeared to be gaining ground rapidly for a few days, when symptoms of effusion in the chest suddenly came on, and in a few days ended her life.

*Dissection.*—A large quantity of purulent fluid was found in the right pleural sac. About half-a-pint of serum was contained in the pericardium, with shreds or flocculi of fibrin, and the left ventricle of the heart was hypertrophied. The kidneys were large and lobulated; and a greyish-white colour, with but little trace of the natural structure, pervaded all the cortical substance. The tubuli were dark, being of a deep chocolate colour.

These cases tend, and in a strong degree, to direct attention to the kidneys in cases of dropsy succeeding scarlatina. There have been hardly enough, at present to establish a conclusion of such consequence.

As we before observed, it is Dr. Barlow's object to shew the efficacy of antiphlogistic and antimonial treatment in the dropsy succeeding scarlatina. The following case is related with that view.

CASE.—*Anasarca and Ascites, consequent upon Scarlatina—Urine highly albuminous—Cerebral Affection,—Complete Recovery.*—William Cowlen, aged three and a half years (Sept. 29, 1835), a fine boy, who has generally been healthy, was five weeks ago the subject of scarlatina, from which he in a great measure recovered, but did not regain his former liveliness and activity. About a week ago he began to swell, first in his face and hands, and afterwards in his whole body; which swelling has continued to increase. About the same time he complained of headache, and became more and more drowsy; and for the last five days his mother has observed his urine to be of a deep dingy red colour. At present he is much swelled over the whole surface of his body; his skin is harsh and dry, but not hot; his eyes nearly closed; tongue covered with a yellowish fur; pupils contracted: the child evinces a great objection to the slightest motion or exertion, and seems to take no notice of any one: pulse sharp, small, and frequent: bowels not open during the last thirty-six hours: urine scanty, of a dingy red colour, and coagulating upon boiling to such a consistence, that it remains in the spoon when inverted.

Pulv. Jalap. C. gr. x. st. sumend.

Ant. Tart. gr. j. Sacchar. purificat. ℥j. M. fiant pulv. xvi. e quibus sumat j. quartâ quâque horâ.—Hirud. ij. pedibus.

Next day he was better, but, on the following, he was worse. He was nearly comatose—the face exceedingly swollen—the pulse small, sharp and very rapid. The bowels had not been opened for twenty-four hours.

Hirud. iij. temporibus.—Pulv. Jalap. C. gr. x. statim.

℞ep. Pulv. tertiâ quâque horâ.

He was much better next day, and continued to improve till the 5th, when the dose of tartar emetic was increased by one-half—two leeches applied to the loins —and a warm bath ordered every night. The perspiration augmented materially under the influence of the latter—the bowels were kept open by the compound jalap powder or by castor-oil—and the dropsical effusion rapidly subsided. The tartarized antimony was gradually diminished in frequency, and the bath used at longer and longer intervals. Under this treatment, his urine gradually lost the dingy-red colour, and regained its natural appearance; its specific gravity also gradually increased, till it reached about 1023; his appetite became very hearty; but animal food was, throughout his convalescence, very sparingly allowed. By the end of November he was, to all appearance, in perfect health; full of flesh, with rosy cheeks, and a clear complexion. His urine, upon the most careful examination, did not afford the slightest evidence of the presence of albumen. Dr. Barlow ascertained that the child continued well in October last, four years after the illness just described.

"I have adduced the above as an instance of a very severe case of this disease following scarlatina, successfully treated by occasional depletion, gentle purgatives, and tartar emetic. I ought to mention, that in a very severe case of the same kind, which occurred to me about a year ago, I used the tartar emetic in larger doses, in proportion to the age of the child; but did not, from the state of its general powers, think it advisable to have recourse to bleeding in any shape; and that the child went on very well, excepting that after the anasarca had disappeared the remedy was continued (the child having been removed into the country) and obstinate diarrhœa ensued, which was, however, at last allayed; and the child ultimately recovered, and was, to my own knowledge, a short time ago, in perfect health."

If it were proved that the dropsy succeeding scarlatina were identical with that resulting from granular degeneration of the kidneys, and if the antimonial treatment just referred to were found to be efficacious for the former, it would, of course, be only reasonable to presume that it would answer well for the latter. But we would observe that men's experience has already established a difference of fact between the scarlatinal and other dropsies, which pathological inquiries can hardly shake—the dropsy after scarlatina has proved *the more curable.* It matters little, at this moment, on what the superior curability may hinge—whether on the patient's youth, or on an essential pathological difference—the fact remains the same, and must preclude any very confident anticipations respecting the effects of remedies on common dropsy.

Dr. Barlow, however, proceeds to apply what he has made out to renal dropsy, and proceeds to detail seven cases, treated on the principles already indicated. The two first we shall pass over, as occurring in children, some doubts may be entertained about them. We shall take:—

CASE.—*Anasarca and Ascites—Albuminous Urine—Complete Recovery.*—"Patk. Queenland, aged 49, Aug. 22, 1839, a stout powerful man, of temperate habits, employed as a porter in a hop-warehouse. A year ago he was under my care for acute rheumatism; since which he had good health, till ten days ago, when he was attacked with headache, and pains in his loins, extending across the epigastrium; ten days afterwards he observed that his feet and scrotum were much swollen, and that his face was puffy in the morning. At present, there is general anasarca, especially of the scrotum, with some ascites.

Pot. Acet. ℈j. Sp. Æth. Nit. ♏xx. Vini Ipecac. ♏x. ex Mist. Mucilag. t. d.

Pil. Scillæ C. Extr. Hyosc. āā gr. v. b. d.

Aug. 24. Little alteration, excepting that he complains of increased thirst. A specimen of urine which I saw to-day gave a copious precipitate with nitric acid: skin dry: pulse hard and sharp: bowels rather torpid.

C. C. lumbis ad x.

Ant. Pot. Tart. gr. ⅛. Mag. Sulph. ʒss. Liq. Ammon. Acet. ℥ss. ex Mist. Camphor. t. d.

Pulv. Ipecac. Comp. gr. v. o. n.

27. Skin still dry: pulse softer: tongue cleaner: pain in the loins much relieved.

Aug. dos. Ant. Pot. Tart. ad gr. ¼.—Pergat.

29. Œdema much reduced: pulse soft: urine less albuminous: slight perspiration at times.

Pulv. Jal. C. ʒss. ault. auroris.

Cont. Mist. sine M.S. et Pulv. ut antea.

Sept. 7. The œdema has entirely disappeared, excepting a little about the ancles: the powders have acted very slightly: no albumen in the urine.

Pulv. Elaterii, gr. ¼. Pulv. Jalap. C. ℈ij. alt. auroris.—Pergat.

12. The last powders have produced rather watery dejections: pulse soft: tongue clean: perspires freely: a little albumen in the urine.

Pulv. Elaterii gr. ⅓. Pulv. Jalap. C. ℈ij. bis in hebdomadâ.

Adde M.S. ʒss. sing. dos. Mist. Cont. Pil.

17. No albumen: no anasarca.

21. Continues well: no albumen.

I have ascertained that this man is now (seven months since his illness) in good health."

The two succeeding cases will be sufficiently explained by their headings, the one being:—*anasarca of two weeks' standing—albuminous urine—recovery apparently complete*—the other,—*scarlatina at the age of eight—anasarca, with albuminous urine, at the age of twenty—recovery complete.* The following remarks of Dr. Barlow's explain his views:—

"The three last cases, I think, afford a proof that the disease under consideration does sometimes make its attack, in the adult, in the form of an acute affection, the time of the invasion of which is well marked; and they further shew, that when thus occurring, and when met sufficiently early with decided treatment, it is susceptible of cure. Of this treatment I believe that the use of tartar emetic forms an essential part:—and I take this opportunity of expressing my opinion of its utility, partly with a view of explaining a statement made by Dr. Bright in the Second Volume of this work, that I had believed it to exert almost a specific influence in this disease. This is, perhaps, more than I ever intended deliberately to affirm, as I much doubt the existence of a specific in any disease whatever. But I believe that, in this instance, the tartar emetic is more;—it is a remedy suggested by the nature of the affection, and calculated to fulfil the most obvious and important indications, namely, equalizing the circulation, subduing the inflammatory action, and restoring the functions of the skin. At the same time, I am far from recommending its use to the exclusion of other means calculated to aid in fulfilling the same indications: and among the most valuable of these adjuncts, I would reckon moderate local depletion, hydragogue cathartics, the warm bath, or, what is perhaps of equal value when this cannot be employed, the investing the loins in a large linseed-meal poultice.

I am aware that a great difference of opinion exists with regard to the diaphoretic plan of treatment in this disease, of which plan the tartar emetic may be regarded as a part; though I am far from regarding it exclusively in this light; —Dr. Osborne having warmly advocated its use; whilst Dr. Christison says, that it disappointed the expectations which he formed from a perusal of the elegant little treatise of the former; and extols the advantages of diuretics, and seems inclined to regard the apprehensions entertained by many, respecting the ill effects which may be expected from their use, as utterly groundless. But, for my own part, I do not think that sufficient stress has been laid by either of these authors upon the different treatment which must be necessarily required by difference in the period and form (whether acute or chronic) of the disease: for I believe that opposite remedies may be useful in its different stages; just as we know that in bronchitis, a disease which bears no very remote analogy to that under consideration, a decidedly antiphlogistic plan of treatment is imperatively called for in the acute and early stage; but that when the inflammatory action has been subdued, or the disease has passed into the chronic form, stimulating expectorants are frequently of essential service, although their inappropriate or too hasty exhibition is attended with the worst results.

Again, it should not be forgotten that copious diaphoresis may occur, either spontaneously or from the effect of remedies, without any concomitant amendment taking place; which happened in the case of Dawkins (case 3 of present communication), and in case 7 of Dr. Bright's communication upon this subject, in the First Volume of this work.

But it is not merely as a diaphoretic that I would recommend the tartar emetic in the acute form of this disease: it is on account of its power of lowering the heart's action, as well as 'its local effects upon the capillaries, when it reaches them through the circulation;' whereby it diminishes the inflammation in the superficial capillaries of the lining membrane of tubuli uriniferi: for that such a state of the tubuli exists in the early stage of the disease is, I think, made apparent by the condition of the kidneys, in all the recent cases which have been examined.

It may be objected, that there is, not unfrequently, so great an irritability of stomach as to preclude the exhibition of the tartar emetic. This state of stomach has, within my observation, been confined mostly to the more-advanced stages of the disease; for I have only met with it in one recent case, which was complicated with extensive peritonitis, involving the peritoneal coat of the stomach.

With regard to the dose of the remedy, I would observe, that where the pulse is hard and full it may be given in such doses as in the first instance to produce nausea; but where there is a low state of the system, the antimony may be given in smaller doses, frequently repeated, so as to reach the capillaries without producing depression. I have never found it necessary to give more than half-a-grain at a dose to an adult; neither have I attempted to push it to the greatest extent possible;—the object not being to give heroic doses of the remedy, but, if possible, to cure the patient."

Setting aside a case in which the albuminous condition of the urine completely disappeared, after two attacks of renal dropsy, we come to the concluding case of Dr. Barlow's, intended to shew, that the plan of treatment recommended above will not be found useless in acute attacks supervening upon the chronic form of the disease, provided a change of measures be adopted as soon as the acute symptoms have subsided; when, as has been already suggested, tonics and moderately stimulating diuretics will be found serviceable.

CASE.—*Albuminous Urine, probably of Two Years' standing—Recent Aggravation—Partial Recovery.*—George Sheppard, aged 49, admitted under Dr. Barlow, at the Surrey Dispensary, July 18th, 1839. He said that he had been for two years gradually losing his strength; and during that time he frequently observed his face to be swollen, especially about the eyelids, when he arose in the morning; and that his legs and ancles often swelled: that a year before he had suffered from general dropsy, attended with pain in the loins and across the epigastrium; for which he was admitted as an out-patient at Guy's Hospital, and obtained considerable relief, though the weakness never ceased entirely: and that about three weeks ago, he was again, after being wet through, attacked in a similar matter.

"At the time I first saw him, his legs, hands, and face, were œdematous, as were the integuments of the loins and abdomen; though I could not ascertain the existence of any fluid in the peritoneal cavity. He complained of aching in the loins, and pain across the epigastrium. His appearance was leucophlegmatic, pulse sharp and frequent, tongue furred, bowels regular. I had not then an opportunity of examining his urine; but having little doubt of the nature of the disease, I ordered him to take one-eighth of a grain of potassio-tartrate of antimony in solution with acetate of ammonia, in camphor mixture, three times daily: five grains of Dover's powders every night: and half-a-drachm of compound jalap powder twice a week: to live chiefly on bread and milk.

On the 20th, I had an opportunity of examining his urine, which was much loaded with albumen. He was then much relieved, and said he had perspired a little on the preceding night."

On the 5th of August, the anasarca had nearly disappeared. He was ordered,

Sp. æth. nit. ♏xx. Tinct. ferri sesquichlorid. ♏xv. ex infus. gentian. comp. t. d.

Pulv. ipecac. c. gr. v. o. n.

Pulv. jalap. c. ʒss. bis in hebdomadâ.

On the 16th of November, he left the dispensary, declaring that he was well, though there was still some albumen in the urine.

These communications of Dr. Bright and Dr. Barlow are both interesting, and calculated to direct still more attention, on the part of practitioners, to complaints so serious and so frequent as these renal affections are.

### ON THE PROPORTION OF UREA IN CERTAIN DISEASED FLUIDS. By G. O. REES, M.D., F.G.S. Physician to the Northern Dispensary.

Dr. Rees' object is to determine the *proportion* of urea contained in the blood in certain morbid states—the *existence* of it being now established. The following is his plan.

"The delicacy of the plan I now adopt is very great; so much so, that urea can be obtained perfectly pure from an animal fluid, which contains it in the proportion only of 0.15 per mille. The analysis is performed as follows:—The serum, or effused fluid, is evaporated to dryness, at a heat sustained somewhat below 212° Fahrenheit; the dry mass is broken up; boiling water thrown upon it, and allowed to digest several hours. This liquor being carefully poured off, a second portion of water is added, and allowed to digest; after which, the whole is thrown on a filter, and the solid matters washed with distilled water till the percolating fluid ceases to effect a solution of nitrate of silver. The digested and filtered liquors are next evaporated to dryness, by a gentle heat; and the extract, so obtained, digested in a stopper-bottle, with common ether of the shops, of sp. grav. 0.754. This menstruum extracts the urea only; and by digesting successive portions of it until the last added yields no deposit of that principle on evaporation, we obtain the whole of the urea present, and thus directly estimate its weight. As obtained by this process, urea is quite pure and colourless. It once happened to me to observe some slight contamination of the urea, obtained as above, by fatty matter which had escaped separation with the albumen: this, however, was easily got rid of, by dissolving the urea in distilled water, and throwing the solution on a filter previously moistened, when the fatty matter remained behind, and allowed the urea to pass through, perfectly pure."

The following quantities of urea were obtained from different specimens of blood.

"The first fluid which I shall mention, as examined by this process, was obtained from John Gillmore, Dec. 18, 1839, a case of albuminuria. It was an effusion on the brain, and 210.4 grs. were obtained for examination. From this quantity 0.05 gr. of urea was obtained, equal to about 0.415 per mille. This fluid yielded but slight traces of albumen.

The second case, from which I obtained the serum of the blood, and likewise a portion of fluid which was procured from the cellular tissue by making punctures in the scrotum, was that of John Wiseman, a boy in Job's Ward, under the care of Dr. Bright. The serum of this patient was of sp. gr. 1015, and contained only 23.49 gr. of albumen in 500 grs.; whereas the same quantity of healthy serum affords about 39.75 grs. It contained 0.2096 per mille of urea. The fluid obtained from the scrotum was of sp. gr. 1007.9: the analysis of 500 parts gave,

| | |
|---|---|
| Water .. .. .. .. .. .. .. | 492.400 |
| Albumen .. .. .. .. .. .. | 0.800 |
| Extractives and salts .. .. .. | 6.725 |
| Urea .. .. .. .. .. .. .. | 0.075 |
| | 500.000 |

the proportion of urea being equal to 0.150 per mille.

The third case, from which I obtained the serum of the blood at two different times, and also the fluid effused into the pericardium, was that of Susan Smalling, a patient of Dr. Bright.

The first specimen of blood was received on March 4th. The specific gravity of its serum was 1025, being somewhat lower than natural: the analysis of 500 grs. yielded,

| | |
|---|---|
| Water .. .. .. .. .. .. .. | 452.10 |
| Albumen .. .. .. .. .. .. | 32.50 |
| Extractives and salts .. .. .. | 15.15 |
| Urea .. .. .. .. .. .. .. | 0.25 |
| | 500.00 |

We here observe a deficiency of albumen, an increase of extractives and salts, and the presence of an ingredient foreign to the serum. The second specimen of blood which I received from this patient was obtained by cupping; and not from the arm, as was the case with all the previously-mentioned specimens. It was sent to me on the 30th of April. The specific gravity of its serum was then 1029, or natural: the analysis of 500 parts was as follows:—

| | |
|---|---|
| Water .. .. .. .. .. .. .. | 448.3 |
| Albumen .. .. .. .. .. .. | 40.8 |
| Extractives and salts .. .. .. | 10.65 |
| Urea .. .. .. .. .. .. .. | 0.25 |
| | 500.00 |

The effusion into the pericardium of this patient was of sp. grav. 1028. It yielded urea, but only a trace in 500 grs. of the effusion.

It will be observed, from the analysis quoted above, that the largest proportion of urea which I have detected in the blood is 0.5 per mille, and the least 0.2096. The effusion on the brain gave 0.415; the fluid effused into the cellular tissue of the scrotum, 0.150 per mille; and the pericardial fluid merely a trace in 500 grains.

The condition of the blood in the patient Susan Smalling is worthy of consideration, inasmuch as the serum of the blood underwent a great change between the dates of March 4th and April 30th: it being sp. grav. 1025 on the former, and 1029 sp. grav. on the latter date: the proportion of urea, however, remaining the same. The serum of the 30th April, if we except the existence of urea, may be considered as normal; the albumen being present quite to the natural extent; indeed, if any thing, somewhat beyond the amount generally found in the serum of healthy blood.

The case from which the scrotal fluid was obtained, affords an instance of great decrease in the specific gravity of the serum of the blood. The lowest specific gravity mentioned by Dr. Christison, in his lately-published valuable work, is 1019; this specimen was, however, only 1015 sp. gr."

Dr. Rees has been unable to obtain urea from the serum of healthy blood.

---

### OBSERVATIONS ON THE EXISTENCE OF CERTAIN ELEMENTS OF THE MILK IN THE URINE DURING UTERO-GESTATION; AND ON THE APPLICATION OF THIS FACT TO THE DIAGNOSIS OF PREGNANCY. By GOLDING BIRD, M.D. F.L.S. &c.

This indefatigable and talented young physician, had his attention drawn by the Medical Journals to a peculiar mucilaginous principle, said to exist in the urine of pregnant women. This new constituent, he says, of the renal secretion, which has been dignified with the name of *Kiestein,* is stated to exist in the urine of the human female during utero-gestation, and to become visible when the secretion is allowed to repose in a cylindrical vessel, in the form of a cotton-like cloud, which in a lapse of time, varying from the second to the sixth day of exposure, becomes resolved into a number of minute opaque bodies, which rise to the surface, forming a fat-like scum, which remains permanent for three or four days: the urine then becomes turbid, and minute flocculi detach themselves from the crust, and sink to the bottom of the vessel: this action continues until the whole pellicle disappears. This crust of Kiestein is stated to be distinguishable from analogous pellicles which occasionally form on the surface of urine, from its never becoming mouldy, or remaining on the surface beyond three or four days from the time of its complete formation.

Dr. Bird accordingly examined the urine first passed in the morning by about thirty pregnant women, and, in all, save three, copious fat-like pellicles were observed on it, after two or three day's exposure. The next thing Dr. Bird did, was to examine the nature of this *Kiestein.*

"None of the specimens of urine voided by pregnant women, that I have examined, were coagulable by heat, nitric acid, or, with but two or three exceptions, by acetic acid, and therefore could not contain any considerable portion of albuminous or caseous matter: the addition of ammonia almost invariably produced a dense deposit of earthy phosphates; which, under the microscope, appeared to consist of myriads of minute acicular crystals; and, with the exception of this proof of the existence of an excess of earthy phosphates in the secretion, no appreciable portion of any anormal ingredients could be detected.

Some of the fat-like pellicle was removed from the surface of some urine on which it had formed, by plunging a plate of glass perpendicularly into the fluid, and withdrawing it adroitly, in a nearly horizontal position; an equable layer of the substance was thus procured; and, when carefully covered with another plate of glass, it could be very conveniently submitted to examination.

The pellicle thus procured, appeared glistening with a lustre like that of spermaceti: when placed under a microscope, and examined with an object-glass of a quarter-inch focal length, myriads of superb triangular prisms, strongly refracting light, and resolving it into colours, were seem imbedded in a mass of irregular granular matter, mixed with which, might here and there be seen patches of tolerably regular globular bodies. The transparent triangular prisms were soon recognised as the well-known crystals of triple phosphate of magnesia; they were so beautifully distinct, and their angles so sharply defined, that the whole became a most interesting microscopic object: some of the crystals were placed on end, and thus appeared like triangular plates."

When the urine is kept so long that the pellicle begins to break up, it, as before stated, falls, in the form of a deposit, to the bottom of the vessel; and if the supernatant fluid be decanted, and the deposit collected on a slip of glass, it is found to present the same appearance as the pellicle; excepting that the crystals are much more numerous, and all the animal matter present is entirely composed of amorphous granules; all trace of any thing like a regular structure being lost.

A slip of glass, on which a portion of the peculiar pellicle had been collected, was placed under the microscope, and covered with a few drops of acetic acid; instantly the whole became opaque, the crystals became rapidly dissolved, and a white pultaceous mass resulted. On washing the whole with a few drops of water, and carefully drying the residue, a white opaque layer was left upon the glass, in which no trace of crystalline matter was perceptible, upon very minute microscopic investigation. Even upon illuminating the object with a beam of polarized light, and analysing the transmitted ray by placing over the eye-glass of the microscope a very fine pale-brown tourmaline, not a trace of colour could be detected; a very faint depolarizing action of the animal matter left on the glass being alone perceptible.

Another portion of the pellicle, also collected on a glass plate, was placed under the microscope, and a few drops of strong liquid ammonia were added: the crystals underwent no change, but became much more distinct from the opaque matter, in which they were imbedded, undergoing solution. In the course of half-an-hour, the glass was carefully washed with a little water, and again examined; when every trace of animal matter was found to have disappeared, and the crystals of the triple phosphate were alone left.

The beautiful crystals present in these pellicles become extremely manifest when a portion of the fat-like scum is collected on a slip of glass, and dried: a drop of Canada-balsam is then placed upon the dried residue; and the whole being gently warmed, a slip of glass, or mica, is pressed flat upon the balsam. When cold, the specimen may be preserved for any length of time. Under the microscope, the crystals are visible with remarkable distinctness; the animal matter mixed with them becoming nearly invisible, from its being immersed in a medium of nearly the same refractive power.

"From the investigations I have made, the greasy aspect of the pellicle of the so-called *Kiestein* arises not from the presence of fat, but from the numerous crystals of triple phosphate, which, from their brilliancy, produce this glistening appearance: with regard to the nature of the animal matter mixed with these crystals, it is difficult, in the present state of physiological chemistry, to give a positive opinion: it is not mere albumen or casein, although much closer allied to the latter than to any other product of organization I am acquainted with, especially when we connect with its chemical character the powerful cheese-like odour so frequently evolved, during its development in the urine, in the form of a pellicle. To this view may be objected the circumstance, that the urine yielding it, does not coagulate on the addition of acetic acid: this, however, is by no means an important objection, as milk, when considerably diluted with a saline solution, or even water, is not perceptibly troubled by acids; so that whilst it may be fairly considered as constituted of an imperfect caseous matter, mixed with crystals of the ammoniacal phosphate of magnesia, it is equally obvious that nothing can be more absurd than to dignify this mixture with a peculiar name, or to consider it as constituting a new organic principle.

There are few products formed during repose in urine which can be readily confounded with this caseous pellicle; if it be borne in mind, that the secretion remains faintly acid up to the moment of breaking up of the crust; which phænomenon I am inclined to regard as arising from the development of ammonia in the urine, as at this time it acquires distinct alkaline properties. The crust of earthy phosphate, which forms on the surface of all urine by long repose, cannot be mistaken for the pellicle under consideration; as that which destroys the latter, viz. putrefaction, causes the production of the former."

After noticing the contradictory opinions of physiologists as to the presence of certain ingredients of the milk in the urine, Dr. Bird remarks,—

"On reviewing the foregoing remarks, and finding, as I believe we do, sufficient evidence of the presence of certain ingredients of the milk, as caseous matter, and abundance of earthy phosphates, in the urine of pregnant women, I would suggest, as a probable explanation opposed to no physiological views that I am acquainted with, that during utero-gestation certain ingredients of the milk are eliminated from the blood by the mammary glands, and, as is very well known, often accumulated in the breasts, in sufficient abundance, to escape from the nipple on pressing it between the fingers. This imperfectly formed secretion not having a ready exit by the mammæ, is taken up into the circulating mass, is separated by the kidneys, and, eventually, escapes from the body in the urine. This view is analogous to the hypothesis of Prof. Burdach, before referred to; and although not quite consonant with the views of M. Rayer, yet is quite in accordance with what we find occurring, under certain circumstances, in the bile, in the cases of obstruction of the biliary ducts; and more rarely in the urine, when, from the presence of calculi or other causes, the ureters are completely obstructed."

Dr. Bird has not met with these pellicles in the renal secretion of nurses, whilst suckling. He mentions a case, which, he thinks appears to justify the idea, that, whilst suckling, the milk being got rid of almost as quickly as it is secreted, none of its elements find their way into the urine; but as soon as the milk ceases to be removed in this way, indications of it are to be met with in the urine, provided pregnancy exists.

He has several times examined the urine of women shortly after their confinement, and hitherto has not succeeded in detecting any indications of the presence of milk in that secretion.

"For want of a sufficient number of observations, I am unable to state how long after conception the urine assumes the properties characteristic of pregnancy. In one case, that of a woman who considered herself to be at the end of the second month of her pregnancy, the urine yielded a well-marked pellicle: but I do not place much confidence in this observation, as the woman might very likely err in calculating how far she was advanced in utero-gestation. As a test for the existence of pregnancy, the formation of the caseous pellicle, especially if accompanied by a cheese-like odour, will, I have no doubt, be an extremely valuable *corroborative* indication: but I would not found on it alone any positive opinion, because, as a sufficient number of observations have not yet been made on this subject, we have no right to assume, however probable it may be, that a caseous pellicle can appear *only* when pregnancy exists. It should be borne in mind, that in many specimens of diabetic urine, as has long ago been shewn by Dr. Aldridge of Dublin, and Dr. Brett of Liverpool, a true deposit of casein, mixed even with traces of butter, occasionally exists. Connected with other symptoms, I should regard the formation of the caseous pellicle in the urine as a valuable corroborative indication; but if existing alone, and unsupported by any other indications of pregnancy, we have no right, in the present state of our knowledge, to regard it as conclusive."

